# Supplementary material for: Integrating genomic structural equation modeling and experimental validation to unravel the genetic basis of male genital lichen sclerosus
Source: Front Immunol. 2026 Jul 16;17:1857945. doi: 10.3389/fimmu.2026.1857945 (PMC13435250; doi:10.3389/fimmu.2026.1857945)
Supplement: Supplementary file 6 [file SupplementaryFile5.zip › result/fuma_PSYCH_factor_sCCA3_focus.chr6.chr6.28917608.29737971.pop1.pdf]

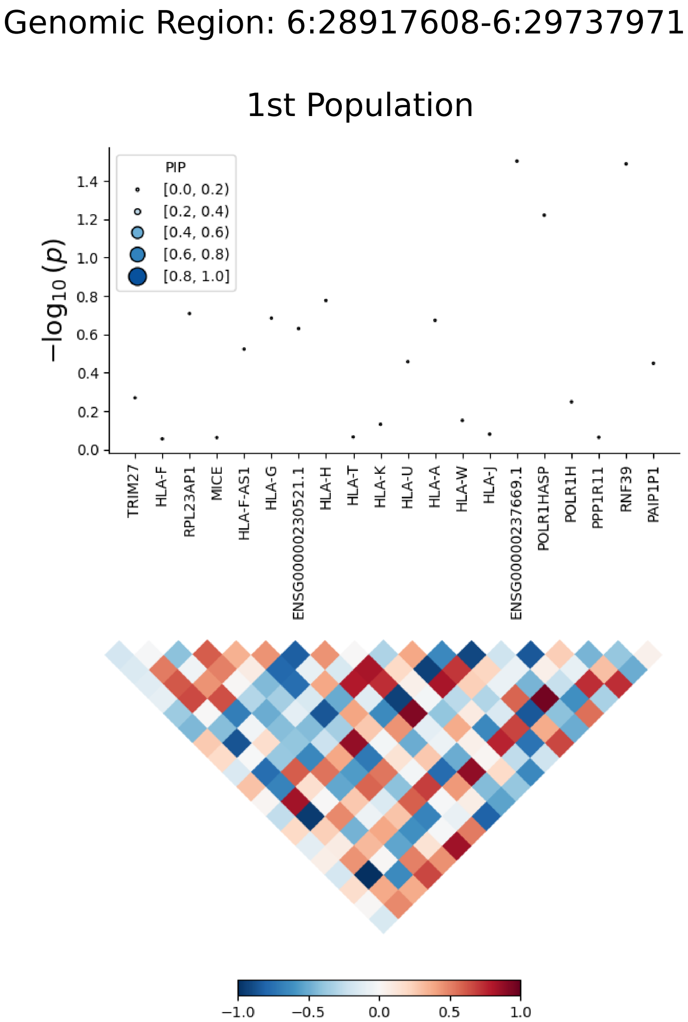
Genomic Region: 6:28917608-6:29737971
1st Population
PIP
[0.0, 0.2)
[0.2, 0.4)
[0.4, 0.6)
[0.6, 0.8)
[0.8, 1.0]
$-\log_{10}(p)$
0.0
0.2
0.4
0.6
0.8
1.0
1.2
1.4
TRIM27
HLA-F
RPL23AP1
MICE
HLA-F-AS1
HLA-G
ENSG00000230521.1
HLA-H
HLA-T
HLA-K
HLA-U
HLA-A
HLA-W
HLA-J
ENSG00000237669.1
POLR1HASP
POLR1H
PPP1R11
RNF39
PAIP1P1
−1.0
−0.5
0.0
0.5
1.0
